# Fructose Metabolism and Its Effect on Glucose-Galactose Malabsorption Patients: A Literature Review

**DOI:** 10.3390/diagnostics13020294

**Published:** 2023-01-12

**Authors:** Nawaf W. Alruwaili, Fahad Alshdayed

**Affiliations:** Department of Community Health Sciences, College of Applied Medical Sciences, King Saud University, Riyadh 11433, Saudi Arabia

**Keywords:** glucose–galactose malabsorption, fructose malabsorption, SLC5A1, SGLT1, metabolic syndrome, autosomal recessive genetic defects

## Abstract

Glucose-galactose malabsorption is a rare inherited autosomal recessive genetic defect. A mutation in the glucose sodium-dependent transporter-1 gene will alter the transportation and absorption of glucose and galactose in the intestine. The defect in the SGLT-1 leads to unabsorbed galactose, glucose, and sodium, which stay in the intestine, leading to dehydration and hyperosmotic diarrhea. Often, glucose-galactose malabsorption patients are highly dependent on fructose, their primary source of carbohydrates. This study aims to investigate all published studies on congenital glucose-galactose malabsorption and fructose malabsorption. One hundred published studies were assessed for eligibility in this study, and thirteen studies were identified and reviewed. Studies showed that high fructose consumption has many health effects and could generate life-threatening complications. None of the published studies included in this review discussed or specified the side effects of fructose consumption as a primary source of carbohydrates in congenital glucose-galactose malabsorption patients.

## 1. Introduction

Carbohydrates are one of the three core nutrients the human body needs in our daily diet, besides fat and protein. Carbohydrates are a cover term that contains essential food groups such as fruits and vegetables, dairy products, fibers, and legumes. It makes up about 45% to 65% of our calorie intake [1,2,3]. Carbohydrate metabolism takes place in different sites throughout the human body systems. The digestion starts in the mouth with salivary amylase. Then, through the gastrointestinal tract, monosaccharides are absorbed into the bloodstream, and the reaction causes the blood glucose levels to increase and some hormones to be secreted, like insulin. Carbohydrate malabsorption consists mainly of two types. The first one is caused due to a rare inborn genetic defect; the second type is related to a pathological condition. Carbohydrate malabsorption can be detected via a hydrogen exhalation test [4]. Metabolic disorders affect the nature of the life of the patients also the patient’s families in many ways, including the social life by being limited to a specific diet and losing the ability to consume meals regularly, and being unable to share meals with others. The cost of all types of therapies may affect the economic status, and the life binding of the patients may require a permanent change in the diet at a high price.

The psychological level of the patient and the patient’s family may be affected due to the patient’s altered lifestyle, the fear of further negative progression of the condition, and frequent visits to healthcare facilities. In some cases, the patient may require staying for long periods admitted to the hospital, which will be stressful for both the patient and the family. Glucose (C_6_H_12_O_6_) is the simplest carbohydrate in the world, and all living organisms are highly dependent on this monosaccharide. Glucose is the human body’s essential energy source in aerobic and anaerobic cellular respiration [1]. Human bodies consume other forms of sugar besides glucose, such as galactose and fructose (monosaccharides), lactose and sucrose (disaccharides), or starch (polysaccharides). Glucose can be stored in the human body as a glycogen polymer which can be used in a time of need energy can be provided through a process called glycolysis which is based on the utilization of glycogen back to glucose. Another process of glucose synthesis is gluconeogenesis [5]. It is the process of generating glucose from non-carbohydrate sources such as fat and protein. Those reactions occur in the fasting state. This shows us the importance of glucose to the human body and its vital role in maintaining homeostasis [1].

Glucose transporters have many differences like tissue type, affinity, and capacity, but the central aspect of difference is the reliability of sodium. There are two main types of glucose transporters. The first one is sodium-dependent glucose transporter-1 (SGLT-1), using the active transport method, and the second type is sodium-independent glucose transporter (GLUT) which uses facilitated diffusion [6]. Fructose is one of the three simple monosaccharides. Among all other natural carbohydrates, fructose has the sweetest taste; thus, it is used as a sweetener in many food products. GLUT5 (SLC2A5) is the primary fructose transporter required to absorb fructose in the intestine [7]. Usually, fructose gets out of the cytosol and enters the bloodstream with GLUT2, a high-capacity, low-affinity transporter [8]. High fructose consumption had some effects on health, and some studies showed that high fructose consumption showed increased hepatic insulin resistance [9]. Also, high fructose consumption showed increased hepatic lipogenesis, which leads to more cardiometabolic effects [10].

Galactose is a monosaccharide found in dairy products in the form of lactose. It is a disaccharide formed of a condensation reaction between glucose and galactose, linking them together to form lactose; galactose is transported into the enterocyte by SGLT-1 and transported out to the bloodstream by GLUT2 [6]. One of the rare inherited autosomal recessive genetic defects is a mutation in the gene of SGLT-1 that will alter the transportation and absorption of glucose and galactose in the intestine and can be detected in the early days of life is responsible for neonatal deaths in many countries [11]. Studies show the prevalence of glucose-galactose malabsorption (GGM) cases in the Arabic region [12]. Parents of many cases were found to be related by blood, which can be related to inheriting the heterogeneous gene responsible for GGM [13,14]. Watery diarrhea symptoms that patients will suffer from when the feeding starts. Also, dehydration is common among patients with GGM, and usually, fail to thrive state is common among them as hypernatremia. The earlier GGM is detected, the better the patient can avoid complications like kidney injury and nephrolithiasis. Some of the diagnostic methods of CGGM are somewhat limited, and the application of these diagnostic procedures is not available in all countries. GGM can be diagnosed through blood sugar levels and sugar chromatography tests, which show glucose and galactose in the stools [15]. Most patients responded positively when the formula was changed to a fructose-based one because GGM patients have normal fructose absorption [16]. The only management that can be applied in avoiding glucose and galactose in the diet and focusing on fructose.

GGM is a rare genetic disorder affecting a few of our populations and is responsible for some deaths in newborns in early life. Most cases are Arabs, especially in Saudi Arabia [12]. Carbohydrate intolerance can result from gastrointestinal malabsorption or, more rarely, from systemic metabolic defects. In gastrointestinal intolerance, a distinction is made between simple and complex carbohydrates. The most common adverse food reactions occur with simple carbohydrates. Clinically, carbohydrate intolerance leads to malabsorption; this occurs in about half of all patients with unspecific food side effects and is often misinterpreted as an allergy. The symptoms of patients with carbohydrate malabsorption are caused by a lack of breakdown or absorption of carbohydrates in the intestinal lumen [4]). Usually, symptoms occur post-feeding, including flatulency, nausea, diarrhea, and unspecific abdominal pain. Occasionally, constipation, weight loss, or extraintestinal symptoms (e.g., fructose malabsorption headache) may be noted. Due to the rapid passage of carbohydrates through the gastrointestinal tract, symptoms often begin as early as 30 min after ingestion; they can stay for 6 to 9 h after eating. The treatment goals for carbohydrate malabsorption are to stop the intake of the responsible carbohydrate substance or decrease it to an amount patients can tolerate [4]. The early detection of the symptoms and the diagnosis of this metabolic disorder are crucial because the earlier detection, the better management can be provided [12].

Congenital glucose-galactose malabsorption (CGGM) is a rare autosomal recessive disorder driven by a galactose and glucose transportation defect across the small intestine [17]. CGGM is an autosomal recessive disorder first defined in 1962 [18]. That usually arises in newborns with severe osmotic-type dehydration and diarrhea [19,20,21]. Diarrhea in CGGM disorder results from the accumulation of unabsorbed galactose and glucose in the lumen of the intestinal leading to delayed growth and development and severe malnutrition [22]. Through the molecular analysis process, one of the responsible genes is solute carrier family 5 member 1 (SLC5A1), with a genetic mutation in chromosome 22q13.1 responsible for this defect. SLC5A1 gene mutation generates the nonfunction of SGLT-1, which is primarily expressed in the intestine brush border membrane [23,24,25]. It was estimated that there are around 300 cases known worldwide. The incidence differs in various populations due to the rarity of occurrence, with some increase in certain areas with higher rates of consanguinity, which supports the CGGM autosomal recessive mode of inheritance [12,19,21,26,27,28].

The defect in the SGLT-1 leads to unabsorbed galactose, glucose, and sodium, which stay in the intestine, leading to dehydration and hyperosmotic diarrhea [29,30]. Thus, the expected characteristics of CGGM are severe dehydration, diarrhea, and weight loss. The best therapy for CGGM is feeding a fructose-based formula. Nevertheless, if the CGGM patient could not be diagnosed and treated immediately, there might be a need for a bowel biopsy and total parenteral nutrition. Eventually, this might lead to severe infection due to malnutrition and long-term total parenteral nutrition [31].

Further complications might occur as well, such as kidney injury, nephrolithiasis, and hypercalcemia [32,33]. Luckily, punctual therapy and precise diagnosis are lifesaving, and the child will be able to grow well into adulthood. Thus, when healthcare providers suspect CGGM, they must diagnose it quickly and accurately [15]. The numerous expected clinical indications of CGGM documented in other studies include abdominal distension, diarrhea, vomiting, and dehydration. Some studies report that CGGM disorder could be inaccurately diagnosed due to hematuria and polyuria. Common symptoms are highly categorized, and the way of CGGM diagnosis is also not specified. Consequently, it is difficult for healthcare providers to reflect on CGGM disorder as their initial diagnosis. This study aims to investigate all published studies on congenital glucose-galactose malabsorption and fructose malabsorption.

## 2. Materials and Methods

A literature review of published studies in PubMed, Google Scholar, ScienceDirect, Saudi Digital Library and the King Saud University library was conducted from inception up to October 10, 2022, using an algorithm based on relative keywords such as “glucose-galactose malabsorption”, “fructose consumption”, “metabolic syndrome”, “carbohydrates malabsorption”, “diarrhea”, “SLC5A1”, “SGLT1”, and “autosomal recessive genetic defects”.

**Figure 1:**
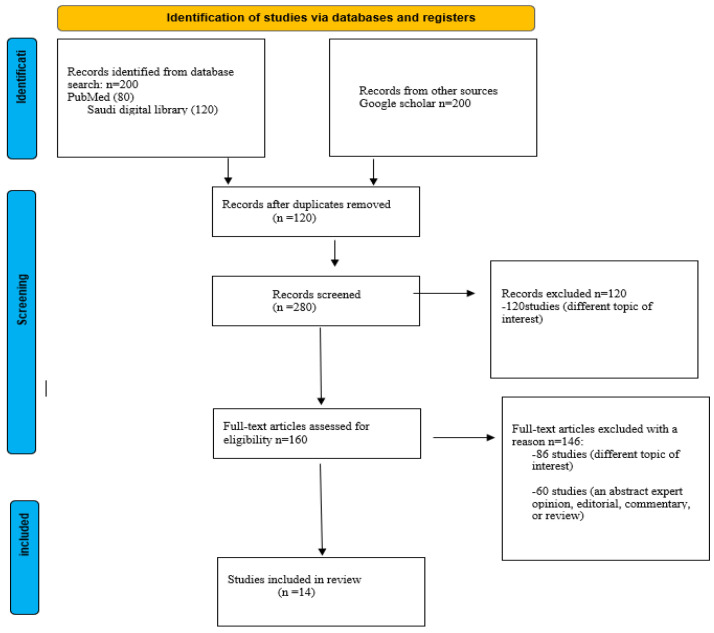
A flow chart showing the screening process of the literature search.

Inclusion criteria include studies that examine the molecular basis and diagnostic methods of CGGM. Also, clinical and personal characteristics of confirmed cases of CGGM, including diagnosis, complications, treatment, country of birth, and nationality of the cases diagnosed. Although, the onset of diarrhea after birth, evidence of carbohydrate malabsorption with a positive test, reducing substance in the stools, and failure to improve lactose-free and amino acid-based elemental formula given in sequence. In addition, a remarkable improvement in diarrhea after the elimination of glucose and galactose using the fructose-based formula; recurrence of diarrhea following exposure to glucose or lactose. Also, articles that examined fructose consumption and its complications were included in this study. Exclusion criteria include chronic diarrhea and non-English studies. Figure 1 shows the process of the screening process of the literature review.

## 3. Results and Discussion

Few published studies have been conducted partially on related interest research. As shown in Table 1, the goals of the included studies have varied from genetics testing studies, investigating fructose consumption on health, understanding the mechanism of fructose metabolism, understanding the effects of excessive sugar intake, examining the laboratory, and reporting the clinical characteristics of patients with CGGM. Also, the goals of included studies vary from examining the association between fructose consumption and isolated slow coronary flow (SCF) and its potential pathophysiological effects. In addition, they aimed to identify whether nuclear receptor LXR takes the fructose transporter GLUT5 (SLC2A5), describing the various aspects of carbohydrate malabsorption and examining the characteristics of patients with CGGM. Furthermore, they aimed to identify the rare Gln457Arg (Q457R) variant on GGM.

Over the last decades, clinical research and epidemiological studies have aimed to report the laboratory and clinical characteristics of patients with CGGM and reduce malnutrition risk factors in the targeted populations. Several factors have been reported addressing the effect of the complications of CGGM.

In a literature review that included 107 CGGM cases, 47 (43.9%) were from Saudi Arabia, 34 (31.8%) were from the United States of America, 9 (8.4%) were from Turkey, 3 (2.7%) were from China, 2 (1.9%) were from the United Kingdom, 2 (1.9%) Canada, and 2 (1.9%) Japan. One case for each was reported from Jordan, Morocco, India, Egypt, Belgium, Macedonia, Iran, and Oman (0.9% for each). Dehydration was stated in all cases, diarrhea was observed in 104 cases (104/107, 97.2%), vomiting was reported in 11 cases (11/107, 10.3%), inadequate weight gain was occurred in 86 cases (86/104, 80.4%), abdominal distension was stated in 51 cases (51/107, 47.7%), fever was occurred in 3 cases (3/107, 2.8%), hematuria/polyuria was observed in 7 cases (7/107, 6.5%), and hypernatremia was stated in 57 cases (57/107, 53.3%). The CGGM complications include kidney injury, hypercalcemia, and nephrolithiasis, which were observed in 20 cases (20/107, 18.7%) [15]. In some cases, polyuria was misdiagnosed [34,35,36]. These symptoms aligned with what was reported in congenital diarrheal disorders (CDD) [37]. Also, it was reported that bilateral renal stones were observed in patients with CGGM [38]. Bilateral renal stones were reported in association with impaired renal function. Chronic diarrhea causes dehydration due to concentrated urine as a potential mechanism. Moreover, bilateral nephrocalcinosis was also associated with renal tubular acidosis in CGGM patients [33,34,39]. Other studies found that CGGM is caused by a mutation in the gene of SGLT1 that will alter the transportation and absorption of glucose and galactose in the intestine. Recently, it was reported that the diagnosis of CGGM can be confirmed by mutational analysis of SLC5A1 [40,41,42].

The SGLT-1 defect results in unabsorbed galactose, glucose, and sodium, which remain in the intestine, causing dehydration and diarrhea. In humans, several factors regulate the transcription of SGLT1 at the promotor region. For example, the binding of hepatic nuclear factor (HNF) 1, transcription factor SP1, and cAMP response element–binding protein (CREB) to the 5′ region of the SGLT1 gene and their impacts on transcription have been documented [43,44].

The studies of the isolated pedigree of GGM patients confirmed the autosomal recessive mode of inheritance. Thirteen GGM cases were added to the pedigree and traced back to the late 17th century. They found that the insertion of the (Q457R) protein into the plasma membrane is impressive as other mutations lead to trafficking defects between the endoplasmic reticulum and the membrane among GGM cases. The failure to translocate bound glucose across the membrane is related to the glucose transport defect by the (Q457R) [13,14]. The using of fructose-based formulas is considered an effective treatment for CGGM cases [15]. A study to evaluate monosaccharides’ absorption rates in three infants with CGGM found that CGGM cases had normal fructose absorption rates [16]. Increased fructose intake can increase serum uric acid levels, which also plays a vital role in developing metabolic disorders, excess energy storage, and weight gain; serum uric acid can be a valuable marker of metabolic disorders and other parameters [45].

A retrospective study was conducted in the western Province of the Kingdom of Saudi Arabia. The study included 24 CGGM patients with a median age of 4.5 months. Half of the subjects were males, 66.7% were Saudi 33.3% were non-Saudi (3 Asians and 5 Arabs). They amid to report the clinical and laboratory characteristics of CGGM patients. They found that 37.5% had affected siblings with CGGM, and 87.5% of the parents were consanguineous. The common symptom was diarrhea and dehydration. Renal stones and nephrocalcinosis were reported in 3 (12.5%) CGGM patients at eight months, twelve months, and seven years, respectively. Renal tubular acidosis and hypernatremia were reported in 16.6% and 29.2%, respectively. During the follow-up, the median follow-up duration was 41.6 months. All except three revealed normal weight gain. Five patients reported one or more symptoms of diarrhea (*n* = 3), bloating (*n* = 3), and abdominal discomfort (*n* = 1). Few children from this cohort persisted in having symptoms of sugar malabsorption during the follow-up. All the subjects had normal development, and none of them had neurological disorders or complications. It was well reported that subjects with CGGM develop symptoms even in maturity with little glucose exposure. The hydrogen breath test stays positive despite thriving well on the fructose replacement formula [27].

Some evidence supports the hypothesis that dietary fructose contributes to the metabolic abnormalities that underpin many chronic diseases. A study was conducted to examine the fibroblast growth factor 21 (FGF21) among participants who were healthy adults aged 18–60 years, with a BMI ranging from 19 to 39 kg/m^2^ without significant medical problems. The study showed a relationship between cardiometabolic risk factors, including obesity, nonalcoholic fatty liver diseases, type 2 diabetes, insulin resistance, and increased FGF21 circulation. Fructose ingestion strongly and acutely stimulates circulating FGF21 [46,47]. Moreover, FGF21 is a liver-derived hormone that controls glucose, lipid, and energy homeostasis and may also experience a feedback mechanism controlling macronutrient selection [45,48,49]. In humans, the serum levels of FGF21 were examined in healthy and overweight subjects to comprehend physiology better. Fatty liver disease is usually associated with being overweight; the increased levels observed with rising body mass index (BMI) might reflect fat accumulation in the liver [50,51,52,53]. Accordingly, the serum levels of FGF21 and hepatic mRNA expression were examined in patients with nonalcoholic fatty liver disease (NAFLD) or nonalcoholic steatohepatitis (NASH). It was reported that the serum levels of FGF21 decreased in individuals with normal weight [46].

A published study focusing on leptin resistance found that leptin resistance developed silently without detecting biochemical or physiological differences between the fructose and control groups and that a high fructose diet promotes obesity. Also, the effects of chronic fructose consumption can develop silently without any metabolic syndrome and visible signs of elevated leptin levels [54]. When fructose is consumed in elevated concentrations on a continuous basis, fructose can promote pathological metabolic changes that may contribute to chronic inflammation, oxidative stress, hyperuricemia, alterations in metabolic hormones, and metabolic syndromes. The role of dysregulated fructose metabolism in chronic disease was examined. The study results showed that excess fructose intake results in high insulin resistance, hyperlipidemia, fatty liver, obesity, and hyperuricemia. The slow coronary flow and its relation to high fructose intake were examined, and results showed a correlation between the slow coronary flow group and higher fructose consumption [55]. Hepatic insulin resistance is associated with the early stages of diabetes development [56,57]. A systematic review and meta-analysis of 46 comparisons in 1005 participants showed that both isocaloric fructose consumption and hypercaloric fructose consumption induce hepatic insulin resistance in normal-weight, nondiabetic adults. This study explicitly implicates fructose is more harmful than other carbohydrates, even over the short to medium term, concerning hepatic insulin sensitivity [58]. This indicates the adverse health effects that fructose metabolism has in such cases.

There are some consequences to high fructose consumption, known as fructose malabsorption. Fructose malabsorption is caused by an overload of the intestinal transport capacity for fructose, distinguishing it from the rare hereditary fructose intolerance (mutation of the aldolase-B gene) [56,59]. The clinical presentation is primarily irritable bowel syndrome with flatulence, abdominal cramps, osmotic diarrhea, and other impaired symptoms. The defective or overloaded transport system in the small intestine is the cause of these symptoms. Therefore, fructose which is not absorbed in the small intestine goes to the large intestine in high concentrations, where the microbiome metabolizes it into hydrogen, methane, carbon dioxide, and short-chain fatty acids [60,61].

**Table 1:** A summary of all articles included in the review *.

Study	Results	Conclusion
[15]	Cases were from Kingdom of Saudi Arabia (55 cases) and Turkey (43 patients) (Total 78.2%) and they were consanguineous marriage. Positive responds when using fructose-based formula. SLC5A1 gene mutations were found in 73 cases (68.2%) after gene testing.	Two major mechanisms in detecting and diagnosing CGGM is fasting and gene testing. The use of fructose-based formulas is the best management for CGGM cases.
[7]	GLUT5 is regulated by LXRα in both mice and humans.	The treatment of metabolic disease and cancer as well LXRα might provide novel pharmacologic strategies for the selective modulation of GLUT5 activity.
[10]	Reduce the intake of SBBS several strategies and policies are needed.	Increased risk of CVD, weight gain, and NIDDM (type 2 diabetes mellitus) with the consumption of SBBS.
[4]	Non-immune- mediated food intolerance has a cumulative prevalence of 30% to 40%, while true (immune-mediated) food allergies affect only 2% to 5% of the German population.	Eliminate the intake of the responsible carbohydrate substance or reduce it to a fair amount might improve the malabsorption.
[13]	Mutant protein is inserted into the plasma. In a duodenal biopsy from a GGM patient, the mutant protein is in the brush border membrane of enterocytes.	Autosomal recessive mode of inheritance was confirmed by the studies of this isolated pedigree of GGM patients, and this provides unique insights into the molecular mechanism of glucose transport by SGLT-1.
[16]	Absorption rate of glucose and galactose is 13% and 22%, respectively. The control group was 85% for glucose and galactose.	There was no difference in the rate absorption of fructose in the control group (healthy individuals) and the study group (three CGGM patients). the rate was 66%.
[45]	The role of excessive intake of fructose and SSBS in developing cardiometabolic diseases was supported by epidemiological data and mechanistic data.	Holistic approaches to bear on present metabolic epidemics to better understand the underlying pathways through which sugar and fructose can cause disease.
[12]	Five patients reported one or more symptoms of bloating (*n* = 3), diarrhea (*n* = 3) and abdominal pain (*n* = 1) during follow up. All had normal development, and none had neurological complications secondary to dehydration.	Early detection and management are crucial might prevent the consequences of dehydration and death resulting from this condition.
[8]	Describes how dietary fructose can affect the expression and activity of fructolytic enzymes and transporters by affecting the cellular response to fructose in the gut.	Dietary fructose may have a potential relation to the gastrointestinal and gut microbe.
[56]	Children, with their preference for sweet foods and drinks, are prone to excessive sugar consumption. Toddlers under age two are especially vulnerable.	The effects that have been observed with the consumption of large amounts of fructose cannot be reliably distinguished from the effects of a generally excessive caloric intake.
[55]	Logistic regression analysis demonstrated that a higher level of Hs-CRP, fructose consumption, and smoking were independently associated with SCF.	The SCF group demonstrated a higher level of fructose consumption. Excessive fructose consumption may play a role in SCF pathophysiology.
[58]	An energy-matched (isocaloric) exchange of dietary carbohydrates by fructose promoted hepatic insulin resistance but had no effect on fasting plasma insulin concentrations. Hypercaloric fructose raised fasting plasma insulin concentrations.	Short-term fructose consumption, in isocaloric exchange or in hypercaloric supplementation, promotes the development of hepatic insulin resistance in nondiabetic adults without affecting peripheral or muscle insulin sensitivity.
[14]	In 22 out of the 23 missense mutations tested in the oocyte expression system, the proteins were translated and were stable in the cell but did not reach the plasma membrane. In four of these mutants, an alanine residue was replaced by a valine, and in two, the trafficking defect was rescued by changing the valine to cysteine. One mutant protein (Q457R) did reach the plasma membrane, but it was unable to transport the sugar across the cell membrane.	Mutations in the SGLT-1 gene are the cause of glucose-galactose malabsorption, and sugar transport is impaired mainly because the mutant proteins are either truncated or are not targeted properly to the cell membrane.

* Abbreviations: SLC5A1, solute carrier family 5 member 1; GLUT5, glucose transport protein 5; SBBS, sugar sweetened beverages; CGGM, congenital glucose-galactose malabsorption; LXRα, liver X receptor alpha; CVD, cardiovascular disease; NIDDM, Non-insulin-dependent diabetes mellitus; GGM, glucose galactose malabsorption; SGLT-1, is sodium-dependent glucose transporter-1; Hs-CRP, high-sensitivity C-reactive protein; SCF, slow coronary flow; Q457R, gln457Arg.

## 4. Conclusions

Despite the considerable heterogeneity of published literature, including several further limitations reported herein, CGGM plays a crucial role in the malnutrition status and clinical outcomes, therefore conducting necessary studies and analyzing its relation to fructose malabsorption management would facilitate a proper nutritional intervention targeting the best nutrition, and typical development of CGGM patients.

Although there are health threats to the high consumption of fructose, a few subjects in our populations are obliged to depend on fructose as their primary source of carbohydrates in their diet. These individuals are CGGM patients, and their suffering from this metabolic disorder is harsh, and its complications threaten their lives. Understanding the metabolism of fructose and the relationship between excessive consumption of fructose and metabolic disturbances provides another opportunity to prevent and treat many chronic diseases. Future studies with adequate power and longitudinal design are nonetheless required to assess further the association of nutritional status with CGGM clinical outcomes using a more standardized definition to measure nutritional status in this population.

## Data Availability

Not applicable.
